# Bacterial and metabolic phenotypes associated with inadequate response to ursodeoxycholic acid treatment in primary biliary cholangitis

**DOI:** 10.1080/19490976.2023.2208501

**Published:** 2023-05-16

**Authors:** Laura Martinez-Gili, Alexandros Pechlivanis, Julie A.K. McDonald, Sofina Begum, Jonathan Badrock, Jessica K. Dyson, Rebecca Jones, Gideon Hirschfield, Stephen D. Ryder, Richard Sandford, Simon Rushbrook, Douglas Thorburn, Simon D. Taylor-Robinson, Mary M.E. Crossey, Julian R. Marchesi, George Mells, Elaine Holmes, David Jones

**Affiliations:** a Division of Systems Medicine, Department of Metabolism, Digestion and Reproduction, Imperial College London, London, UK; b Division of Digestive Diseases, Department of Metabolism, Digestion and Reproduction, Imperial College London, London, UK; c Biomic_Auth, Bioanalysis and Omics Laboratory, Center for Interdisciplinary Research and Innovation (CIRI-AUTH), Balkan Centre, Thessaloniki, Greece; d Academic Department of Medical Genetics, Cambridge University, Cambridge, UK; e Liver Unit, Freeman Hospital, Newcastle Upon Tyne Hospitals NHS Foundation Trust, Newcastle upon Tyne, UK; f Institute of Translational and Clinical Research, Newcastle University, Newcastle upon Tyne, UK; g Leeds Liver Unit, St James’s University Hospital, Leeds, UK; h Center for Liver and Gastroenterology Research and National Institute for Health Research (NIHR) Birmingham Biomedical Research Centre, University of Birmingham, Birmingham, UK; i NIHR Biomedical Research Centre at Nottingham University Hospitals NHS Trust, University of Nottingham, Nottingham, UK; j Department of Gastroenterology, Norfolk and Norwich University Hospital, Norwich, UK; k UCL Royal Free Campus, Royal Free Hospital, University College London Institute of Liver and Digestive Health, London, UK; l Department of Electrical and Electronic Engineering, Imperial College London, London, UK; m Department of Hepatology, Cambridge University Hospitals NHS Foundation Trust, Cambridge, UK; n Center for Computational & Systems Medicine, Murdoch University, Perth, Australia

**Keywords:** Bile acids, gut-liver-kidney axis, microbiota, PBC, UDCA

## Abstract

Primary biliary cholangitis (PBC) is a chronic cholestatic liver disease with ursodeoxycholic acid (UDCA) as first-line treatment. Poor response to UDCA is associated with a higher risk of progressing to cirrhosis, but the underlying mechanisms are unclear. UDCA modulates the composition of primary and bacterial-derived bile acids (BAs). We characterized the phenotypic response to UDCA based on BA and bacterial profiles of PBC patients treated with UDCA. Patients from the UK-PBC cohort (*n* = 419) treated with UDCA for a minimum of 12-months were assessed using the Barcelona dynamic response criteria. BAs from serum, urine, and feces were analyzed using Ultra-High-Performance Liquid Chromatography-Mass Spectrometry and fecal bacterial composition measured using 16S rRNA gene sequencing. We identified 191 non-responders, 212 responders, and a subgroup of responders with persistently elevated liver biomarkers (*n* = 16). Responders had higher fecal secondary and tertiary BAs than non-responders and lower urinary bile acid abundances, with the exception of 12-dehydrocholic acid, which was higher in responders. The sub-group of responders with poor liver function showed lower alpha-diversity evenness, lower abundance of fecal secondary and tertiary BAs than the other groups and lower levels of phyla with BA-deconjugation capacity (*Actinobacteriota*/*Actinomycetota*, *Desulfobacterota*, *Verrucomicrobiota*) compared to responders. UDCA dynamic response was associated with an increased capacity to generate oxo-/epimerized secondary BAs. 12-dehydrocholic acid is a potential biomarker of treatment response. Lower alpha-diversity and lower abundance of bacteria with BA deconjugation capacity might be associated with an incomplete response to treatment in some patients.

## Introduction

Primary biliary cholangitis (PBC) is a chronic cholestatic liver disease, characterized by biliary epithelial cell (BEC) stress, injury, and loss. The condition affects approximately 35 people per 100,000 in the UK,^1^ and lifestyle-related factors like higher BMI have been associated with disease progression.^2–4^ Bile acids (BAs) play a key role in both PBC pathogenesis and treatment; hydrophobic BAs are thought to contribute to BEC stress and injury, and therapy approaches that reduce or modify toxic BAs lie at the heart of current disease management strategies.

The first-line treatment approach for PBC currently is oral administration of ursodeoxycholic acid (UDCA), a hydrophilic secondary BA that improves serum biochemical markers of liver damage and can delay disease progression. UDCA response is not, however, universal and patients with a sub-optimal response need second-line therapies such as obeticholic acid. Despite the key role played by “non-response” to UDCA in treatment strategy decision-making, the reasons for this state of inadequate response have received little attention and the mechanisms are currently poorly understood. The concept of UDCA (non-)response is in itself complex, with a number of identified and validated criteria, based on degrees of serum biochemical markers abnormality after UDCA therapy.^5^ Consequently, many patients deemed to be UDCA responders actually have ongoing serum biochemical abnormalities, albeit at a level below the defined response threshold. This disease stratification in itself has been challenged with recent data suggesting that any ongoing biochemical abnormality in circulating liver markers entails increased risk of progression.^6^

Our ability to treat PBC optimally would be greatly facilitated by an increase in our understanding as to the true nature of both non-response to UDCA and the mechanisms of partial response. While the word “response” has been used to classify patients across different criteria, it is important to distinguish when the goal is to dynamically assess within-patient biomarker levels post-treatment compared to pre-treatment, or when the term “response” relates to assessment of disease prognosis in a population, in which case only post-treatment biomarker levels are considered as part of the criteria. Response criteria that consider within-individual biomarker longitudinal changes, such as the Barcelona or Nara criteria,^7,8^ are particularly suited to understanding the lack of *dynamic* response to treatment, while criteria that do not account for pre-treatment levels, such as the Toronto criteria,^9^ are more suited to predict disease *prognosis*. Due to this subtle but important difference, in this work we will refer to *dynamic* or *prognostic* response criteria accordingly.

Bile acids undergo enterohepatic circulation and are exposed to the gut micro-environment. The contribution of the gut microbiota to cholestatic diseases is being increasingly investigated, as evidence of the strong crosstalk between BA metabolism and gut microbiota mounts.^10,11^ This crosstalk is particularly true in the case of primary sclerosing cholangitis (PSC), for which there is a high co-morbidity with inflammatory bowel disease (IBD).^12^ In PBC, *Pseudomonas* and *Sphingomonas* genera and their corresponding family and order taxa were more abundant in the terminal ileum mucosa of patients treated with UDCA compared to a group without PBC;^13^ fecal bacterial composition was associated with genetic variants of the human leukocyte antigen in a Chinese cohort of patients with PBC;^14^ and one study showed lower bacterial richness and different bacterial composition in patients compared to healthy controls, which was partially reversed by treatment with UDCA.^15^ These findings, while on a relatively small cohort of patients, suggest that further research is needed to uncover within-individual mechanisms of treatment response and assess whether a gut-liver axis could be exploited for a better disease management in PBC.

The UK-PBC cohort, established in 2007, is one of the largest cohorts of PBC patients assembled to date. The cohort remains a unique and important resource to identify new disease risk, progression and treatment response biomarkers that could later be validated in subsequent mechanistic studies.^16^ In previous works, we characterized genomic and serum proteomic signatures associated with disease status and prognostic response criteria.^17,18^ Here, we assess the relationship between BAs and gut bacterial composition in 419 patients of the UK-PBC cohort to investigate differences between UDCA dynamic responders and non-responders.

## Results

### PBC patients respond differently to UDCA

Different static prognostic and dynamic response criteria have been developed for PBC, based on absolute levels or improvement of biomarkers of liver function in blood respectively, which determine the status of response to treatment with UDCA. In this study, we initially focused on the dynamic response to treatment in a UK-PBC sub-cohort of patients affected with PBC and taking UDCA for at least 1 year. Patients with either a decrease in alkaline phosphatase (ALP) >40% of pre-treatment values or normal levels after at least 1 year of treatment with UDCA were classified as responders (R) for the current study (Barcelona criteria).^7^ Patients with ongoing elevation of alkaline phosphatase and who had not shown a 40% reduction with UDCA therapy were classified as non-responders (NR).

**Figure 1.**
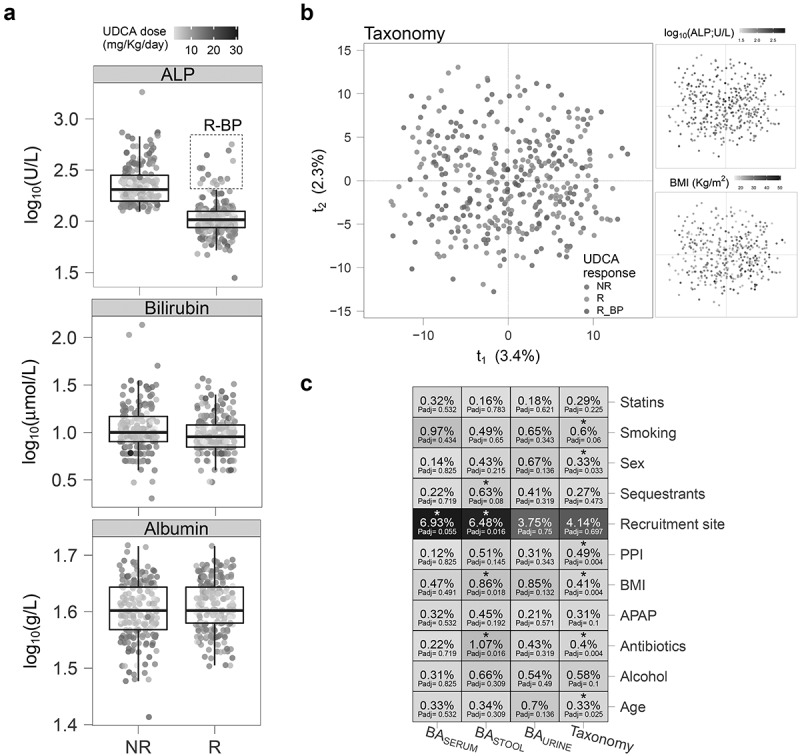
Dynamic response to UDCA treatment varies across patients. a) Box-and-whisker plots of serum markers in non-responders (NR; *n* = 191) and responders (R; *n* = 228) according to the Barcelona criteria and indicating the newly identified subgroup of responders with bad prognosis (R_BP; *n* = 16) with a dashed square. b) PCA scores of clr-transformed ASV abundances (*n* = 380). c) PERMANOVA variation (R^2^) percentage attributed to each factor, with corresponding Benjamini-Hochberg adjusted *P*-value (Padj) indicated within each cell. *n* = 380 taxonomy; 366 serum; 362 feces; 400 urine. ALP: alkaline phosphatase; APAP: acetaminophen (paracetamol); BA: bile acid; PPI: proton pump inhibitor; UDCA: ursodeoxycholic acid.

Application of the Barcelona criteria to our study cohort resulted in identification of 191 non-responders (NR) and 228 responders (R) to UDCA. The responders had significantly lower levels of serum bilirubin (T-test *P* = 0.024), but no difference in serum albumin (T-test *P* = 0.094). Of the 228 responders using the Barcelona criteria, 212 were also responders using the most widely used static “good prognosis” cutoff of an absolute alkaline phosphatase level after UDCA therapy of <1.67× ULN (the threshold for response in the Toronto/POISE prognostic criteria^9^). The remaining 16 showed, however, a positive response in terms of the dynamic criterion (>40% reduction in alkaline phosphatase) whilst their alkaline phosphatase value remained >1.67× ULN putting them, paradoxically, simultaneously also into a bad prognosis group (Figure 1a; see Methods). Interestingly, while there were no significant differences in treatment dose or duration in this group (Table 1), these individuals had lower serum albumin and higher serum alanine transaminase and bilirubin concentrations; features which are associated with worse disease progression,^5^ as well as a higher mean age and a higher proportion of individuals co-treated with bile acid sequestrants. We were interested in exploring the biological basis of this group with an apparently mixed response to UDCA. Given their distinct circulating liver biomarker profile, we named this subset of responders “responders with bad prognosis” (R_BP) and investigated whether their different response to treatment was associated with specific metabolic or bacterial phenotypes.

**Table 1.** Cohort Characteristics.

	Total *N* = 419	NR *N* = 191 (45.6%)	R *N* = 212 (50.6%)	R_BP*N* = 16 (3.8%)	*P*
**Sex N (%)**					
Female	374 (89.3)	172 (90.1)	188 (88.7)	14 (87.5)	0.778
Male	45 (10.7)	19 (9.9)	24 (11.3)	2 (12.5)	
**Age (y)** Mean (SD)	63.42 (10.31)	61.97 (10.69)	64.47 (9.99)	66.81 (7.70)	**0.021**
**Age at diagnosis** **(y)** Mean (SD)	53.39 (10.3)	52.29 (10.72)	54.33 (9.99)	53.93 (7.99)	0.135
**BMI** Mean (SD)	28.13 (6.14)	27.62 (6.12)	28.68 (6.23)	27.01 (4.58)	0.172
**AMA N (%)** Negative Positive	31 (9.0) 312 (91.0)	16 (10.5) 136 (89.5)	13 (7.4) 163 (92.6)	2 (13.3) 13 (86.7)	0.401
**Alkaline phosphatase (U/L)** Mean (SD)	181.73 (144.11)	257.16 (173.14)	104.15 (29.45)	309.31 (109.03)	**<0.001**
**Bilirubin (μmol/L)** Mean (SD)	11.72 (10.00)	13.07 (13.33)	10.41 (5.53)	13.12 (7.85)	**0.018**
**Albumin (g/L)** Mean (SD)	40.59 (4.57)	40.17 (4.85)	41.11 (4.24)	38.62 (4.54)	**0.026**
**Alanine transaminase(U/L)** Mean (SD)	37.84(29.14)	46.69(34.04)	28.89 (20.46)	50.12 (28.13)	**<0.001**
**Platelet count(x109/L)** Mean (SD)	255.24 (97.69)	264.46 (115.78)	250.17 (77.81)	211.50 (81.51)	0.273
**UDCA dose (mg/Kg/day)** Mean (SD)	12.69 (3.56)	13.15 (3.58)	12.30 (3.46)	12.37 (4.19)	0.054
**UDCA (y)** Mean (SD)	9.45 (6.29)	9.17 (6.09)	9.44 (6.40)	12.93 (6.60)	0.076
**Sequestrants N (%)** No	397 (94.7)	178 (93.2)	206 (97.2)	13 (81.2)	**0.018**
Yes	22 (5.3)	13 (6.8)	6 (2.8)	3 (18.8)	
**Antibiotics N (%)** No	339 (80.9)	150 (78.5)	177 (83.5)	12 (75.0)	0.317
Yes	80 (19.1)	41 (21.5)	35 (16.5)	4 (25.0)	
**APAP N (%)** No	395 (94.3)	181 (94.8)	199 (93.9)	15 (93.8)	0.81
Yes	24 (5.7)	10 (5.2)	13 (6.1)	1 (6.2)	
**Statins N (%)** No	356 (85.0)	164 (85.9)	179 (84.4)	13 (81.2)	0.804
Yes	63 (15.0)	27 (14.1)	33 (15.6)	3 (18.8)	
**PPI N (%)** No	288 (68.7)	141 (73.8)	138 (65.1)	9 (56.2)	0.089
Yes	131 (31.3)	50 (26.2)	74 (34.9)	7 (43.8)	
**Alcohol N (%)** Abstinent	140 (33.4)	64 (33.5)	71 (33.5)	5 (31.2)	0.983
Moderate	254 (60.6)	115 (60.2)	128 (60.4)	11 (68.8)	
Excess	25 (6.0)	12 (6.3)	13 (6.1)	0 (0.0)	
**Smoking N (%)** Never	157 (37.5)	77 (40.3)	75 (35.4)	5 (31.2)	0.829
Former	224 (53.5)	98 (51.3)	116 (54.7)	10 (62.5)	
Current	38 (9.1)	16 (8.4)	21 (9.9)	1 (6.2)	
**Autoimmune diseases N (%)** Any	105 (25.1)	40 (20.9)	59 (27.8)	6 (37.5)	0.128
Celiac	8 (1.9)	4 (2.1)	2 (0.9)	2 (12.5)	**0.025**

Significance across treatment response groups was assessed by Fisher’s Exact Test for categorical variables. One-Way ANOVA was used for normally distributed continuous variables (age, BMI, albumin, UDCA dose, and log_10_-transformed alkaline phosphatase, bilirubin, alanine transaminase and platelet count) and Kruskal-Wallis was used for UDCA treatment years. AMA: anti-mitochondrial antibody; APAP: acetaminophen (paracetamol); NR: non-responder; PPI: proton pump inhibitor; R: responder; R_BP: responder with bad prognosis; UDCA: ursodeoxycholic acid.

### Variability of metabolite and gut bacteria profiles

To interpret the data, sources of variability in the datasets were investigated. Unsupervised Principal Component Analysis (PCA) did not show strong clusters according to treatment response or serum ALP concentration (Figure 1b and Supplementary Figure S1), but there was an increasing BMI gradient along the first component for taxonomy and fecal BAs. Patient geographical area was the main source of variation across all datasets (Figure 1c) but was only significant for fecal and serum BAs after adjusting for false discovery rate (FDR; 10% FDR adjusted *P*-value (P_adj_) <0.1). As expected, we identified many sources of variation for bacterial composition: age, sex, BMI, antibiotics, smoking and proton pump inhibitors (PPI), while bile acid sequestrants contributed to the variation in fecal bile acids.

### Fecal and urine bile acids are associated with UDCA response

**Figure 2.**
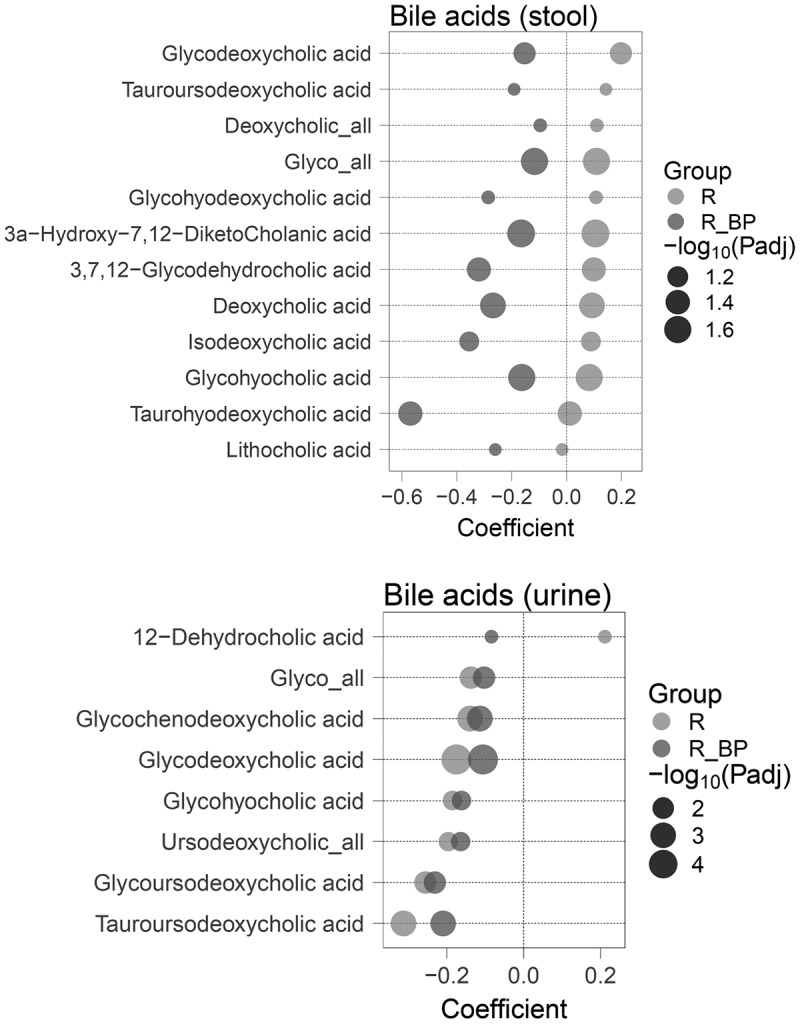
Bile acids in feces and urine differ with UDCA response. Regression coefficients of significantly different fecal (top) and urine (bottom) bile acids according to UDCA response, with non-responders as reference category. *P*-values were determined using a likelihood ratio test of nested models and adjusted using Benjamini-Hochberg, with a 10% false discovery rate threshold (P_adj_<0.1). *n* = 362 feces (163 NR; 184 R; 15 R_BP); *n* = 400 urine (183 NR; 201 R; 16 R_BP). NR: non-responder; R: responder; R_BP: responder with bad prognosis.

First, we compared fecal BA profiles across the three study groups (R, NR and R_BP). The responders showed higher levels of 12 fecal bile acids compared to the non-responders (Figure 2 and Supplementary Table S1). These included the bacterial-derived secondary BAs deoxycholic acid (DCA: β = 0.093; 95% CI [0.0026,0.18]) and lithocholic acid (LCA: β= −0.015; 95% CI [−0.089,0.058]). Taurine-conjugated UDCA (T-UDCA) was significantly higher in responders (β = 0.14; 95% CI [0.015,0.27]), despite there being no significant differences in treatment dose between responders and non-responders (Table 1). One of the mechanisms by which UDCA exerts its beneficial effects is by decreasing BAs intestinal absorption.^19^ We found a higher abundance of glycine-conjugated BAs (G-BAs) summed intensities in the feces of responders (β = 0.11; 95% CI [0.04,0.18]), while total BA relative abundance – comprising the sum of intensities of all annotated conjugated and unconjugated species – remained similar across groups. Interestingly, a completely opposite pattern was seen in the paradoxical R_BP group with - in contrast to the elevated levels of fecal BAs seen in the conventional responders - reduction of all 12 of the bile acids compared to non-responders (DCA: β= −0.27; 95% CI [−0.49,-0.044], LCA: β= −0.26; 95% CI [−0.44,-0.077]) (Figure 2 and Supplementary Table S1).

We also compared fecal BA differences across response groups after adjusting for bile acid sequestrant intake, which we identified as a potential source of variability in the fecal metabolome (Figure 1c). The same trends were still observed in 5 out of the 12 BAs, with a significantly higher abundance of DCA and G-BAs in R, and a lower abundance of the secondary bile acid DCA and tertiary bile acid tauro-hyodeoxycholic acid (an LCA hepatic metabolite) in R_BP with respect to NR (Supplementary Figure S2 and Supplementary Table S1).

In urine, summed conjugated and unconjugated abundance of UDCA, G-BAs and another six BAs, specifically glycine-conjugated forms of the primary bile acid chenodeoxycholic acid (CDCA), DCA and taurine- or glycine-conjugated UDCA, were higher in non-responders than both responder groups. There were no differences in urine creatinine levels across groups (One-Way ANOVA *P* = 0.81; Supplementary Figure S2), indicating that the higher levels of these bile acids in non-responders might be independent of kidney function or other factors that could affect urinary excretion. In contrast to the other assessed bile acids, 12-dehydrocholic acid (12-DHCA) was specifically elevated in the urine of responders compared to non-responders (β = 0.21; 95% CI [0.051,0.37]; Figure 2 and Supplementary Table S2).

Finally, no differences in serum BAs or fecal short chain fatty acids (SCFAs) were found between groups (Supplementary Tables S3 and S4).

### Bacterial composition differs in confounder-matched samples

**Figure 3.**
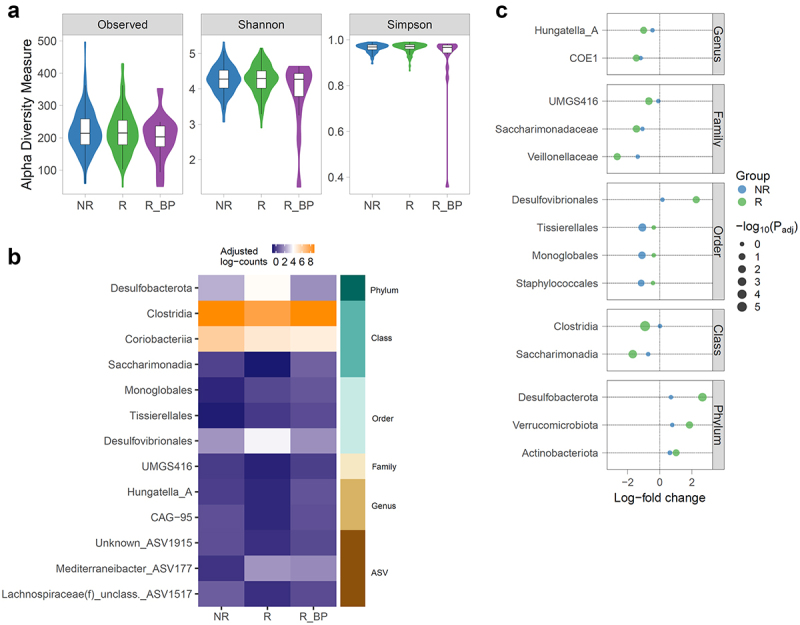
Bacterial differences in unmatched and matched groups. a) Alpha diversity measures in response groups. Richness was not significantly different, while Shannon and Simpson were significantly lower in R_BP. *P*-values were determined using a likelihood ratio test of nested mixed models as specified in Methods, and adjusted using Benjamini-Hochberg, with a 10% false discovery rate threshold (P_adj_<0.1). *n* = 380 (169 NR; 196 R; 15 R_BP). P_adj_ = 0.019 Shannon; P_adj_ = 2.03e-06 Simpson. b) Significant taxa in R_BP-matched subset, determined with ANCOMBC omnibus test. *n* = 42 (14 NR; 13 R; 15 R_BP). Heatmap shows the log-transformed counts adjusted by sampling fraction determined by the ANCOMBC algorithm, with white color corresponding to the overall median of the represented counts. c) Significant taxa in R_BP-matched subset, determined with ANCOMBC pairwise test, with R_BP group as reference category. Values are log-fold change abundances with respect to the reference category. *n* = 42 (14 NR; 13 R; 15 R_BP). NR: non-responder; R: responder; R_BP: responder with bad prognosis.

We identified 9,865 amplicon sequence variants (ASVs), of which only 447 were present in at least 10% of specimens. Responders had a similar alpha-diversity to non-responders, while R_BP had a lower Shannon and Simpson evenness (Figure 3a and Supplementary Table S5).

Differential abundance analysis between response groups was done using ANCOMBC omnibus and pairwise tests.^20^ The omnibus test showed genus *Sellimonas* was differently abundant across the three groups, with highest mean abundance in NR and lowest in R_BP, and the pairwise comparisons identified an order with placeholder name ML615J–28, from the *Bacilli* class, which was significantly lower in R_BP compared to non-responders (Supplementary Tables S6 and S7).

Given the different sources of variability affecting microbial composition identified in our cohort and by others,^21^ and the characteristics of the R_BP group, we applied ANCOMBC on a subset of samples matched by confounders (see Methods), comparing only individuals with similar characteristics (sex, age, BMI, sequestrants, smoking, PPI, antibiotics and hospital) to R_BP (Supplementary Table S8). The omnibus test on the matched subset detected differences in three ASVs, two genera, one family, three orders, three classes and the phylum *Desulfobacterota* (Figure 3b and Supplementary Table S9). Specifically, dynamic responders had the highest abundance of *Desulfobacterota*, its order *Desulfovibrionales* and a *Mediterraneibacter*-assigned ASV, and lowest abundance of *Clostridia* and *Saccharimonadia*, while non-responders had higher abundance of *Coriobacteriia*, and lower abundance of *Monoglobales* and *Tissierellales*. Dynamic responders with bad prognosis (R_BP) had the lowest abundance of *Coriobacteriia* and highest abundance of *Monoglobales* and *Tissierellales*. Pairwise results showed that compared to R_BP, responders also had lower abundance of *Veillonellaceae* family and higher abundance of phyla *Verrucomicrobiota* and *Actinobacteriota* (now named *Actinomycetota*), and non-responders had lower abundance of order *Staphylococcales* (Figure 3c and Supplementary Table S10).

**Figure 4.**
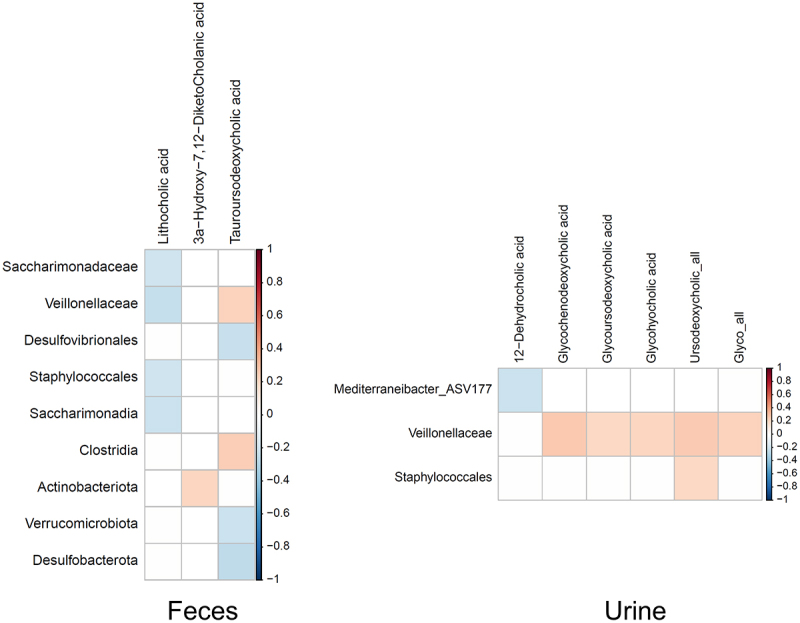
Correlations between bile acids and taxa. Pearson correlations between the identified significant taxa (ANCOMBC-adjusted abundances) and bile acids intensities in feces (right) and urine (left). *P*-values were adjusted using Benjamini-Hochberg, with a 10% false discovery rate threshold (P_adj_<0.1). Only significant correlations with an absolute coefficient value equal or bigger than 0.2 are shown. *n* = 380 (169 NR; 196 R; 15 R_BP). NR: non-responder; R: responder; R_BP: responder with bad prognosis.

Abundance of significant taxonomic features was associated with fecal and urine bile acid intensities differing across response groups (Figure 4). Phyla *Desulfobacterota* and *Verrucomicrobiota*, higher in R_BP-matched responders, anti-correlated with fecal T-UDCA intensity, while *Actinobacteriota* positively correlated with a secondary bile acid derived from cholic acid: 3α-hydroxy-7,12-diketocholanic acid, where the 7-OH and 12-OH groups had been oxidized by action of 7α- and 12α-hydroxysteroid dehydrogenases. Instead, less abundant taxa in matched responders such as *Clostridia*, *Saccharimonadia* or *Veillonellaceae*, positively correlated with T-UDCA and negatively correlated with LCA. Similarly, *Veillonellaceae* correlated with CDCA and UDCA bile acids in urine, while an ASV assigned to the genus *Mediterraneibacter* negatively correlated with 12-DHCA.

## Discussion

Lack of response to UDCA treatment in PBC is associated with a significantly increased risk of progression to cirrhosis, liver transplantation and lower survival. Despite its importance as a clinical challenge, the mechanisms underlying UDCA non-response are poorly understood. We explored metabolic and bacterial differences in a cohort of 419 UDCA-treated patients, 46% of whom had not responded to treatment when assessed using dynamic response criteria. In addition, we identified a subgroup of patients who had responded to treatment according to the Barcelona dynamic criteria, but paradoxically failed to reduce ALP levels to less than 1.67 × ULN (R_BP group), and hypothesized that their phenotype might be different to other dynamic responders. Our data led us to conclude there are relevant metabolic and bacterial differences between UDCA responders and non-responders, suggesting that the gut micro-environment may play an important role in determining the response to UDCA. The responder with bad-prognosis group had, however, a strikingly different phenotype compared to the main responder group. The identification of this group may allow us to dissect out the various components of the process of response to UDCA.

**Figure 5.**
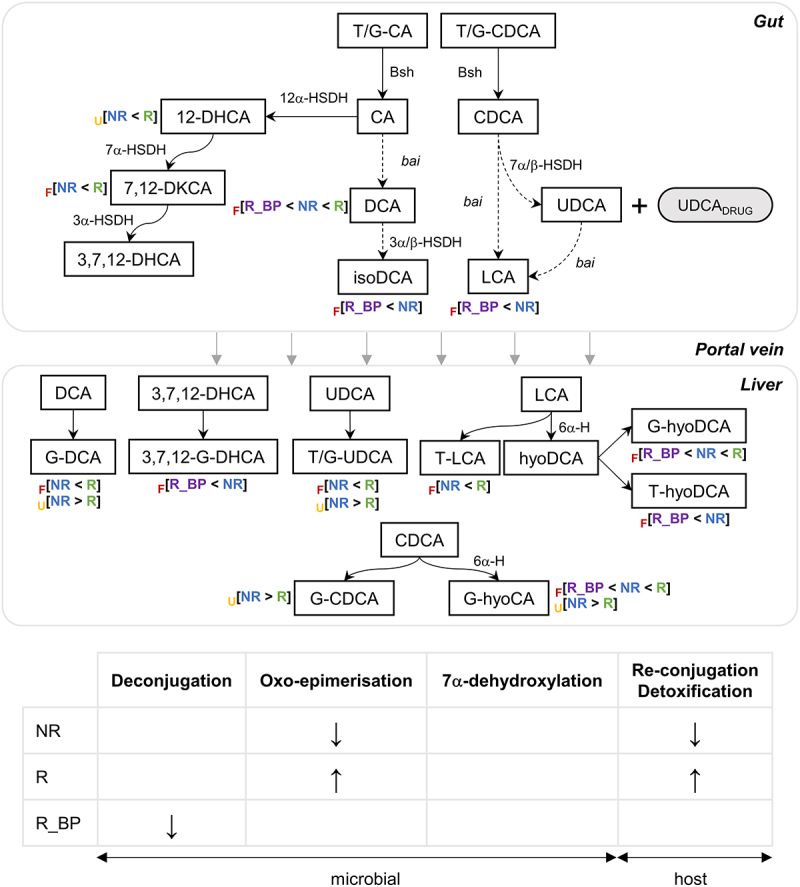
Summary of changes occurring in different treatment responses. Dynamic responders (R) had higher fecal excretion of conjugated secondary and oxo-BAs and increased urine 12-dehydrocholic acid. R_BP had lower excretion of unconjugated secondary BAs. Dashed arrows indicate multi-step reactions. Blank cells in the summary table of proposed bacterial functional differences (bottom) indicate that our data do not provide enough evidence of whether these pathways are different across groups. 6α-H: 6-α hydroxylase (CYP3A4); bai: BA-induced operon enzymes for 7α-dehydroxylation; Bsh: bile salt hydrolase; CA: cholic acid; CDCA: chenodeoxycholic acid; DCA: deoxycholic acid; DHCA: dehydrocholic acid; DKCA: diketocholanic acid; F: feces; HSDH: hydroxysteroid dehydrogenase; LCA: lithocholic acid; NR: non-responder; R: responder; R_BP: responder with bad prognosis; T/G: taurine-/glycine-conjugated; U: urine; UDCA: ursodeoxycholic acid.

Fecal bile acids demonstrated a different signature across responses (Figures 2 and 5). Dynamic responders with bad prognosis (R_BP) excreted less unconjugated secondary BAs DCA and LCA and their downstream derivates such as isoDCA and T-hyoDCA than non-responders, signifying that R_BP might have lower BA deconjugation capacity by bacterial bile salt hydrolases (Bsh), together with lower capacity for 7α/β-dehydroxylation reactions as well (Figure 5). Consistent with this hypothesis, we found lower abundance of phyla that harbor the *bsh* gene in R_BP than R, namely *Desulfobacterota*, *Verrucomicrobiota* and *Actinobacteriota*/*Actinomycetota* (Figure 3). Responders had higher relative abundance of fecal glycine-conjugated BAs and taurine-conjugated UDCA, indicating higher hepatic re-conjugation and excretion, reflective of an improvement of cholestasis and liver function. T-UDCA is a known anti-inflammatory chemical chaperone,^22^ so a higher presence in the liver of responders could contribute to the tissue healing process. In addition, since G-BAs are more hydrophobic than T-BAs (hence considered more toxic for the hepatic parenchyma and biliary ducts^23^), the reduced excretion of G-BAs in NR and R_BP patients could expose them to persistently higher toxic BA species and tissue damage. Another factor indicating higher hepatic detoxification in responders compared to both NR and R_BP, was the increased fecal excretion of the 6α-hydroxyl BAs glyco-hyocholic and glyco-hyodeoxycholic acids, which are CDCA and LCA products respectively, generated in the liver by 6α-hydroxylase (6α-H; CYP3A4; EC 1.14.14.57), a phase I detoxification cytochrome P450 enzyme.^24^

SCFAs are bacterial metabolites produced from dietary sources of indigestible fiber with important roles in inflammation and gut homeostasis.^25^ A recent study found that total fecal SCFAs and acetate were higher in PBC patients with fibrosis with respect to patients without fibrosis,^26^ however, to our knowledge there are no studies that have characterized SCFAs with respect to UDCA dynamic response. We did not find differences in fecal SCFAs across response groups, leading us to conclude that UDCA dynamic response is not associated with differences in fecal SCFA abundances and that their role, if any, might be secondary to the one of the bile acid *milieu* and likely through indirect modulation of gut homeostasis and immune pathways.

The gut-liver-kidney axis has been less often addressed in PBC than the gut–liver connection. The kidney absorbs circulating BAs through the solute carrier family 10 member 2 (SLC10A2; formerly ASBT) and exports them back to the systemic circulation to be re-assimilated by the liver.^27^ Under normal physiological conditions, BAs are found in low concentrations in urine, but cholestasis can result in a buildup of BAs in serum and urine as a compensatory mechanism to avoid their accumulation in the liver parenchyma.^28^ Supporting the evidence of a persistent higher systemic exposure of hydrophobic BAs, we found that non-responders had higher levels of G-BAs, G-CDCA and UDCA in urine compared to responders. However, we did not find any association between serum bile acids and UDCA response. This result is not surprising, as total serum bile acids have historically been reported to remain similar after UDCA treatment – although an increase in UDCA percentage has been observed when comparing serum bile acid composition before and after treatment initiation, which could not be tested in our cohort due to its cross-sectional design -,^29,30^ and it is likely that changes in serum bile acids are only observed in those patients with a more advanced stage of liver disease.^31,32^ Interestingly, 12-dehydrocholic acid (12-DHCA) was the only bile acid specifically increased in the urine of responders, which could imply a higher rate of synthesis and systemic bioavailability in these patients. 12-DHCA is a secondary bile acid produced from cholic acid (CA) by the bacterial enzyme 12α-hydroxysteroid dehydrogenase (12α-HSDH; EC 1.1.1.176), which removes the hydrogen from the 12-OH group. The enzyme has been identified in *Clostridium* and *Eggerthella* species.^33^ In our cohort only 3 ASVs out of 9,865 were assigned to the genus *Eggerthella* and 9 to *Clostridium*: of these, two *Eggerthella* ASVs were present in 30% of the samples and were not differently prevalent nor abundant across response groups, and the rest were present in less than 10% of the samples and were not further analyzed, as there is no method to analyze such sparse features without introducing biases. It is possible that there exist other yet unidentified taxa able to produce 12-DHCA; in agreement with this hypothesis, we found a positive correlation between *Eggerthella*’s phylum *Actinobacteriota*/*Actinomycetota*, higher in R, and 7,12-diketocholanic acid in feces, a 12-DHCA derivate upon which the 7α-OH has been oxidized by a bacterial 7α-HSDH (Figure 5). Another bacterial enzyme that could produce 12-DHCA is 12β-HSDH (EC 1.1.1.238), from 12-epicholic acid, but information on this gene in bioinformatic databases and research publications is also scarce. Further research is needed to ascertain whether 12-DHCA has an active role in the favorable response to UDCA or whether it can be used as an early biomarker of response. Given that modifications by HSDH increase BAs hydrophilicity,^34^ 12-DHCA could have a choleretic effect. Studies supporting this conclusion have been carried out mainly in animals and using the fully oxidized 3,7,12-DHCA.^35,36^

Metataxonomic compositions between R and NR were surprisingly similar given the observed differences in BA profiles, so we postulate that the bacterial contribution to treatment dynamic response relies on the function and activity of the broad bacterial community rather than on few dominant organisms, and that integrative metagenomics, metatranscriptomics, and/or metaproteomics might be needed to fully understand the observed changes in secondary bile acids. This was not the case for R_BP patients, which had reduced alpha diversity evenness compared to NR, and differently abundant taxa compared to matched NR and R. Given the significant associations between taxa and bile acids (Figure 4), we suggest that when it comes to differing dynamic responses to UDCA, the identified taxonomic changes might be relevant for the impaired treatment response observed in some patients (Table 1). BMI is a risk factor for disease progression and is associated with changes in gut microbiome. While we did not find any differences in BMI across response groups after one year of treatment, and taxonomic changes were independent of BMI, future prospective studies should monitor whether any weight gain during treatment can increase the risk of impaired response. Despite the low numbers of patients in the R_BP group, there would be significant clinical benefit in accurately stratifying these patients early in their treatment journey and we advocate for longitudinal studies measuring microbial and metabolic features before and after intervention in bigger cohorts. Interestingly, we observed that baseline ALP concentrations in the R_BP group fell in the upper quartile of the corresponding NR and R distributions (Supplementary Figure S4). However, it is unclear whether this can be a robust stratification criterion for predicting an impaired UDCA dynamic response in the general population. Further studies enrolling higher numbers of participants with clinical similarities to the R_BP group are required to ascertain this.

Bile acid sequestrants are usually prescribed to manage pruritus, a PBC complication.^37^ More patients in the R_BP group (19%) were prescribed bile acid sequestrants compared to NR (7%) and R (3%). We found that sequestrants did not affect bacterial composition in our cohort, but they may affect fecal bile acids composition (Figure 1c). It is important to note that our findings are limited by the fact that only 22 out of 419 patients (5.3%) were co-treated with sequestrants, so further research is needed to confirm whether sequestrants are associated with a higher risk of partial or non-response to UDCA and elucidate their impact on bacterial and metabolic changes. To our knowledge, there is only one recent study on 33 UDCA-treated PBC patients with jaundice, which were given cholestyramine for a total of 16 weeks to assess its effects on bile acids and microbial composition.^38^ The study found that individuals with higher reduction in serum bilirubin at 16 weeks had increased fecal bile acids excretion, serum SCFAs and two *Lachnospiraceae* species (SCFAs producers), compared to baseline. However, it is unclear how these changes ultimately relate to UDCA dynamic or prognostic responses.

Our study has other limitations: our analysis only considered antibiotics taken within the last 3 months prior to sample collection. While many studies show a recovery of microbial diversity one month after a course of antibiotics,^39^ there are considerable inter-individual differences and recent studies have shown long lasting or even potentially permanent changes.^40^ The degree of fibrosis is a contributing factor for PBC progression.^41^ While we did not find differences in fibrosis between NR and R patients (Fisher’s *P* = 0.14), we could not assess this in R_BP, owing to the unavailability of transient elastography records in 19% of patients in that group. The bile acid method did not annotate sulfated bile acid species which are commonly found in feces, serum and urine;^32^ similar to the observed increase in 6α-hydroxylated BAs, it is possible that dynamic responders have higher excretion of sulfated-BAs as well, as they are both detoxifying hepatic modifications. Recently, novel bile acid conjugations carried by gut microbes have been discovered,^42^ so it will be important to investigate what role these species may have in PBC in future studies. We cannot discard the possibility that pre-treatment extant bacteria could influence UDCA response as we could not test this hypothesis with the cross-sectional design of our cohort. It would therefore be important for future studies to assess bacterial and BA differences at baseline in responders compared to partial and non-responders. In this regard, two studies in smaller cohorts have analyzed bacterial changes before and after UDCA treatment. One study found a decrease in taurine-conjugated BAs post-treatment, which was inversely associated with *Bilophila*, consistent with this genus ability to metabolize taurine.^43^ However, conclusions of the study are limited by the lack of an explicit differential abundance analysis on the taxonomy data itself between pre- and post-treatment. The other study found *Veillonella* persistently increased in PARIS II prognostic non-responders.^15^ Although we did not find differences in *Veillonella* in dynamic non-responders, the *Veillonellaceae* family was lower in R than R_BP (Figure 3c), further supporting a role for *Veillonellaceae* family and its members in the outcome of UDCA dynamic and prognostic response.

Overall, our findings show a significant difference in fecal and urine bile acid profiles in UDCA treatment dynamic responders, particularly secondary and tertiary host-bacterial compounds. We identified urine 12-DHCA as a potential novel biomarker of favorable treatment response and showed that reduced alpha diversity evenness and gut bacterial composition are associated with an impaired treatment response. Our results also open the path to test new hypotheses on the mechanistic role of increased 12-DHCA in responders and highlight the need for studies aimed at determining the effect of bile acid sequestrants in treatment response.

## Patients and methods

### Study cohort

The cohort (described in Carbone et al.^16^ consisted of 419 adults (≥18 y old) with PBC who had received UDCA for at least 12 months (Table 1). PBC diagnosis was determined by presence of at least two of the following criteria: a) serum anti-mitochondrial antibodies (AMA) at a titer≥1:40, b) persistently elevated serum alkaline phosphatase (ALP) prior to treatment with UDCA, c) liver histology consistent with PBC. Patients within the eligibility criteria (≥18 y old with diagnosed PBC and receiving UDCA treatment) were recruited across 20 National Health Service (NHS) hospitals in the UK. The study was conducted in accord with the guidelines of the Declaration of Helsinki and the principles of good clinical practice, and was approved by the Oxford C research ethics committee (REC reference: 07/H0606/96) and by the research and development department of each collaborating hospital. All participants provided written informed consent.

### Response classification

To classify patients as UDCA responders, we initially applied the Barcelona criteria.^7^ The set of outliers in the responders’ group ALP distribution (Figure 1a) – viz., values outside the whiskers limits of the box-and-whiskers plot, equivalent to an ALP level beyond the distribution’s 75^th^ quantile plus 1.5 times its interquartile range (IQR)) – coincided with ALP levels higher than 1.67 times the upper limit of normal (ULN), a threshold used by the Toronto criteria associated with bad prognosis.^9^ In addition, we observed that these individuals had lower serum albumin and higher bilirubin than the other responders (Table 1). We therefore defined, for the purposes of the study, three response groups in the cohort: non-responders (NR), responders (R; ALP levels reduced >40% or below the ULN after at least 1 year of treatment) or responders with bad prognosis (R_BP; ALP levels reduced >40% after 1 year of treatment, but still higher than 1.67 × ULN). The ULN for ALP depends on the biochemical assay kit that was used in each hospital, therefore one of the 16 R_BP patients does not appear as an outlier in Figure 1a.

### Sample collection and storage

Blood and urine samples were collected on-site during the clinical visit at the assigned collection centers (Newcastle, Leeds, Birmingham, Nottingham, Cambridge, Norwich, London: Imperial College Healthcare, Royal Free). All collection centers used the same materials and followed strictly the same standard operating procedures (SOPs) for sample collection, handling, storing, and shipping. Briefly, blood was left to clot for 1 hour at room temperature (RT) to obtain the serum and second urine void of the day was placed on ice. Samples were centrifuged (1,000 ×g; 10 min; 4°C) and supernatant divided into aliquots. Stool samples were collected by the patient with a FecesCatcher® 48 hours prior to the appointment, transferred into sterile tubes and frozen at −20°C in a domestic freezer. Patients brought the frozen fecal sample in a provided cooler bag with a U-Tek® pack the day of the visit. All samples were stored at −80°C until further processing.

### Ultra-high-performance liquid chromatography-mass spectrometry bile acid profiling

#### Sample preparation

*Fecal extracts*. Fecal samples were freeze-dried upon arrival at the Imperial College metabolomics facility. 100 mg were extracted using bead-beating with 1 mL of solvent (2:1:1, H2O:IPA:ACN) or 500 µL, if <50 mg were obtained, and the extracts were split for the subsequent metabolomic assays. 80 µL of fecal extract were transferred to a well in a 96-well plate and 20 µL used to generate the quality control (QC) samples.

*Serum and urine*. Serum and urine samples were thawed and centrifuged (16,000 ×g; 20 min; 4°C). 50 µL of serum were mixed with 150 µL of cold methanol, followed by incubation at −20°C for 24 hours and 75 µL of urine were diluted with an equal volume of IPA:ACN (1:1). Tubes were vortexed and centrifuged (16,000 ×g; 10 min; 4°C), 100 µL of supernatant were transferred to wells in a 96-well plate and 10 µL used to produce the pooled QC sample.

*Quality control samples*. QC samples were prepared by pooling equal volumes of each study sample into a single tube and divided into aliquots. QC samples were placed at the beginning and end of the run and interspersed evenly with the randomized study samples. In addition, they were spiked with mixtures of 56 BA standards (Steraloids, Newport, RI) to determine their chromatographic retention times.

#### Data acquisition and processing

Ultra-High-Performance Liquid Chromatography coupled with Mass Spectrometry (UHPLC-MS) was performed using the method described in Sarafian *et al*.,^44^ with an Acquity UPLC column coupled to a Xevo G2 Q-ToF mass spectrometer (Waters Ltd, Elstree, UK) equipped with an electrospray ionization source operating in negative ion mode. Waters Ltd raw files were converted to .mzML format with the msConvert tool from ProteoWizard software.^45^ BAs were annotated using the retention time of the spiked standards as reference and peaks area integrated using *peakPantheR* R-package.^46^ Features below the limit of detection (LOD) in more than 90% of QC samples were discarded. To adjust for signal intensity decay along the run, each feature was divided by a smoothed curve fitted on the QC’s total ion intensity, generated with the loess function from the *stats* R-package (package specification will be omitted for functions within *stats* beyond this point). Normalized features with a coefficient of variation greater than 30% in QC samples or below LOD in more than 20% of study samples were discarded. For fecal samples, relative intensities were further normalized by mg of fecal material used. All features were log-transformed and missing values imputed using impute.QRILC from the *imputeLCMD* R-package. For statistical analyses, features were also mean-centered.

### Proton nuclear magnetic resonance spectroscopy

Relative intensities of fecal SCFAs and urine creatinine were obtained using untargeted Proton Nuclear Magnetic Resonance Spectroscopy (^1^H-NMR). Briefly, 100 µL of fecal extract (80 µL when extracted with 500 µL of solvent) were lyophilized and the residue resuspended in 600 µL of LC-MS-grade H_2_O. 540 µL of the fecal supernatant or thawed urine samples were mixed with 60 µL of NMR buffer prepared with D_2_O (1.5 M NaH_2_PO_4_, 5.8 mM 3-(Trimethylsilyl) propionic-2,2,3,3-d4 acid sodium salt (TSP; SIGMA, UK), 2 mM NaN_3_, pH 7.4), centrifuged (12,000 ×g; 4°C; 5 min), and placed in a 5 mm SampleJet NMR tube (Bruker, Germany) for ^1^H-NMR spectroscopic analysis. QC samples were prepared by pooling equal amounts of every study sample into a single tube and dividing it into 600 µL aliquots in separate tubes. QC samples were analyzed simultaneously with the randomized study samples and evenly spread across the run. ^1^H-NMR experiments were carried out using a Bruker Avance spectrometer (Bruker, Germany) operating at 600 MHz as described previously.^47^ Spectra were acquired through a standard 1-dimensional pulse sequence using the first increment of the Nuclear Overhauser Effect (NOE) pulse sequence to achieve water suppression, and 2D J-resolved (JRES) spectra for aiding metabolite identification. Spectra were imported into MATLAB using in-house scripts. Redundant spectral regions (water peak and flanking empty peak areas) were removed and data normalized by probabilistic quotient normalization (PQN).^48^ Features were annotated using statistical total correlation spectroscopy (STOCSY)^49^ and ^1^H-NMR spectra and peak databases. Area under the curve (AUC) of a representative peak was calculated for each annotated feature and used for statistical analysis.

### 16S rRNA gene sequencing

DNA was extracted from 250 mg of fecal samples using PowerLyzer PowerSoil DNA Isolation Kit (Mo Bio, Carlsbad, CA, USA) following manufacturer’s instructions, with the addition of a bead-beating step for 3 min at speed 8 in a Bullet Blender Storm (Chembio Ltd, St Albans, UK). DNA was stored at − 80°C. Sample libraries amplifying the V1–V2 region of the 16S rRNA gene were prepared following Illumina’s 16S Metagenomic Sequencing Library Preparation Protocol and as described previously.^50^ Sequencing was performed in two batches on an Illumina MiSeq platform using the MiSeq Reagent Kit v3 (Illumina Inc, San Diego, USA) and paired-end 300-bp chemistry. Data were demultiplexed, barcodes removed and FASTQ files generated using Illumina software BCL Convert. Amplicon sequence variants (ASVs) were generated using *DADA2* R-package;^51^ for each batch, primer sequences and 3’-end nucleotides were trimmed using the filterAndTrim function with maxEE= Inf, to avoid introducing bias during the quality filtering step.^52^ ASVs from each batch were then merged and chimeras removed. Taxonomy was assigned with the IdTaxa function from *DECIPHER* R-package,^53^ and using the Genome Taxonomy Database release 95 (GTDB_r95) as a training set, provided in the package website (http://www2.decipher.codes/Downloads.html). Presence of contaminant sequences was assessed with isContaminant from *decontam* R-package.^54^ Twelve contaminant sequences were detected and removed, two of them commonly found in DNA reagent kits (*Rhodococcus* spp and *Herbaspirillum* spp). To build a phylogenetic tree, sequences were aligned using Clustal Omega algorithm with the *msa* R-package and a maximum parsimony tree was built with the function pratchet, from the *phangorn* R-package using default parameters. Faith’s phylogenetic diversity (PD) was calculated using *picante* R-package. For statistical analyses, except for α-diversity calculations, features with a zero count in more than 90% of samples were discarded, left-censored zero values were imputed with cmultRepl from *zCompositions* R-package,^55^ using a geometric Bayesian multiplicative (GBM) replacement method,^56^ and data were centered-log-ratio (clr)-transformed.

### Statistical methods

Principal Component Analysis (PCA) was performed with the opls function from the *ropls* R-package.^57^ When measured ASV counts are clr-transformed, PCA can be used to inspect beta-diversity.^58^ Permutational Multivariate Analysis of Variance (PERMANOVA) was used to quantify the variance explained by each factor in Figure 1c. Euclidean distance matrices were built for each normalized omics dataset with the function dist and used as input in the adonis2 function of the *vegan* R-package. Variance corresponds to the total variance explainable by that variable, as it was calculated independently of other variables (that is, the factor was the only predictor in the model). For missing records in categorical variables (*N* = 12 (2.9%) antibiotics; *N* = 11 (2.6%) APAP; *N* = 8 (1.9%) smoking), we imputed the most common category. Given the collinearity between weight and BMI (Spearman *P* = 0.9), missing BMI records (*N* = 10 (2.4%)) were predicted by linear regression with weight and sex as explanatory variables using lm. When weight was also missing (*N* = 3 (0.7%)), sex-specific BMI median values were imputed.

Bacterial differential abundance (DA) significance testing was performed with *ANCOMBC* ^20^ using the following per-feature model:

feature ~ response + sex + age + BMI + antibiotics + PPI + smoking + hospital

where *response* had three categories: non-responders (NR; reference group), R and R_BP (see response classification). Antibiotics and PPI were binary variables indicating whether patients took antibiotics within the last 3 months or proton pump inhibitors (PPI) regularly. Smoking history had three categories: “never,” “former,” and “current.” Hospital acronym, with 20 categories, was included as a proxy of geographical area to account for the different location of the recruited participants. Continuous variables (age and BMI) were mean-centered and univariance-scaled. ANCOM first estimates features with structural zero values across experimental groups,^59^ which are considered statistically significant. Features not found to be consistently absent in one of the experimental groups were tested for abundance difference across groups. However, we estimated that in our dataset, at least 50 samples per group are needed to detect structural zeros with an acceptable false-positive rate (see Supplementary Notes), and therefore the function parameter struc_zero was set to FALSE. *P*-values were adjusted (P_adj_) using the Holm method and null hypothesis was rejected if P_adj_<0.1.

For metabolomics data, the same model was fitted with lmer (*lme4* R-package^60^, with hospital as a random intercept instead of fixed effect, and *response* significance assessed with a likelihood ratio test (anova R function) *versus* a null model with the same co-variates, but without the *response* variable. *P*-values were adjusted with p.adjust using the Benjamini-Hochberg method and P_adj_<0.1 considered significant. Adjusted *P*-values are denoted as “P_adj_” throughout the text regardless of method used.

To generate the R_BP-matched subset, a Euclidean distance matrix was first created using sex, age, BMI, sequestrants, smoking, PPI, antibiotics and hospital as variables. Categories were first converted into integers and all variables were mean-centered and univariance-scaled. For each R_BP sample, a NR and R sample with the minimum distance value was selected. Non-imputed BMI was used for matching, and sample size across groups was not artificially balanced by iteratively removing each match from the selection population. This generated 12 matched R and 15 matched NR samples (Supplementary Table S8). The R_BP-matched metataxonomic dataset was analyzed with *ANCOMBC* as previously described, however due to the reduced sample numbers only age and BMI were added as covariates in the model.

Correlations between omics were performed with corr.test from the *psych* R-package and the heatmap was plotted using *corrplot* R-package. In addition, *tidyverse* ^61^ and *ggpubr* R-packages were used to generate the figures.

## Supplementary Material

Supplemental Material

## Data Availability

16S rRNA gene amplicon sequences have been deposited in the European Nucleotide Archive database with reference PRJEB44791. Data sharing will be considered upon reasonable request. https://www.ebi.ac.uk/ena/browser/view/PRJEB44791

## Abbreviations

6α-H: 6-α hydroxylase (CYP3A4)
ACN: acetonitrile
ALP: alkaline phosphatase
AMA: anti-mitochondrial antibodies
APAP: acetaminophen (paracetamol)
ASV: amplicon sequence variant
AUC: area under the curve
BA: bile acid
*bai*: bile acid inducible operon
BMI: body mass index
Bsh: bile salt hydrolase
CA: cholic acid
CDCA: chenodeoxycholic acid
CI: confidence interval
DA: differential abundance
DCA: deoxycholic acid
DHCA: dehydrocholic acid
DKCA: diketocholanic acid
EC: enzyme commission
F: feces
G-BA: glycine-conjugated bile acid
GBM: Geometric Bayesian Multiplicative
HSDH: hydroxysteroid dehydrogenase
IPA: isopropanol
IQR: interquartile range
LCA: lithocholic acid
LOD: limit of detection
MS: mass spectrometry
NMR: nuclear magnetic resonance
NR: non-responder
P_adj_: adjusted P-value
PBC: primary biliary cholangitis
PCA: principal component analysis
PPI: proton pump inhibitor
PQN: probabilistic quotient normalisation
PSC: primary sclerosing cholangitis
QC: quality control
R: responder
R_BP: responder with bad prognosis
RT: room temperature
SCFA: short-chain fatty acid
STOCSY: statistical total correlation spectroscopy
U: urine
ULN: upper limit of normal
UHPLC: ultra-high-performance liquid chromatography
