# Supplementary material for: Bacterial and metabolic phenotypes associated with inadequate response to ursodeoxycholic acid treatment in primary biliary cholangitis
Source: Gut Microbes. 2023 May 16;15(1):2208501. doi: 10.1080/19490976.2023.2208501 (PMC10190197; doi:10.1080/19490976.2023.2208501)
Supplement: Supplemental Material [file KGMI_A_2208501_SM5085.zip › KGMI_2208501_Supplemental/Supplementary_Notes.pdf]

Supplementary Notes

The ability to identify ASV as structural zeros in study groups - denoting truly absent taxa rather than insufficient sequencing depth - is a powerful tool, as it could indicate rearrangements in bacterial community structure. However, the inherent sparsity of metataxonomy data, together with the smaller sample size in the R_BP group, could lead to overestimation of absent taxa in R_BP. Indeed, while none of the 447 ASV were found to be absent in NR and R groups, 253 and 264 ASV respectively, were declared structural zeros when using the same number of samples as in R_BP, matched by confounders ("R_BP-matched"; see Methods; **Supplementary Figure 3A**). Since ANCOM does not provide any measure of certainty when determining structural zeros, we calculated the false positive rate (FPR) of declaring taxa as truly absent in our cohort depending on the number of specimens sampled, and estimated that with 16 samples, 50-55% could be false positives (**Supplementary Figure 3B**).

False positive rate calculation. False positive rate for identifying structural zeros at different fixed sample sizes (*n*), was computed by randomly selecting *n* samples from NR and R groups and running ANCOM pre-processing to identify number of detected structural zeros. FPR was calculated as:

$$FPR = \frac{number\ of\ strutural\ zeros\ detected\ (false\ positives)}{total\ number\ of\ ASV\ (true\ negatives + false\ positives)}$$

Results from 100 random permutations for each sample size were plotted using geom_smooth, from *ggplot2* R-package.
