# Supplementary figures and images for: Bacterial and metabolic phenotypes associated with inadequate response to ursodeoxycholic acid treatment in primary biliary cholangitis
Source: Gut Microbes. 2023 May 16;15(1):2208501. doi: 10.1080/19490976.2023.2208501 (PMC10190197; doi:10.1080/19490976.2023.2208501)

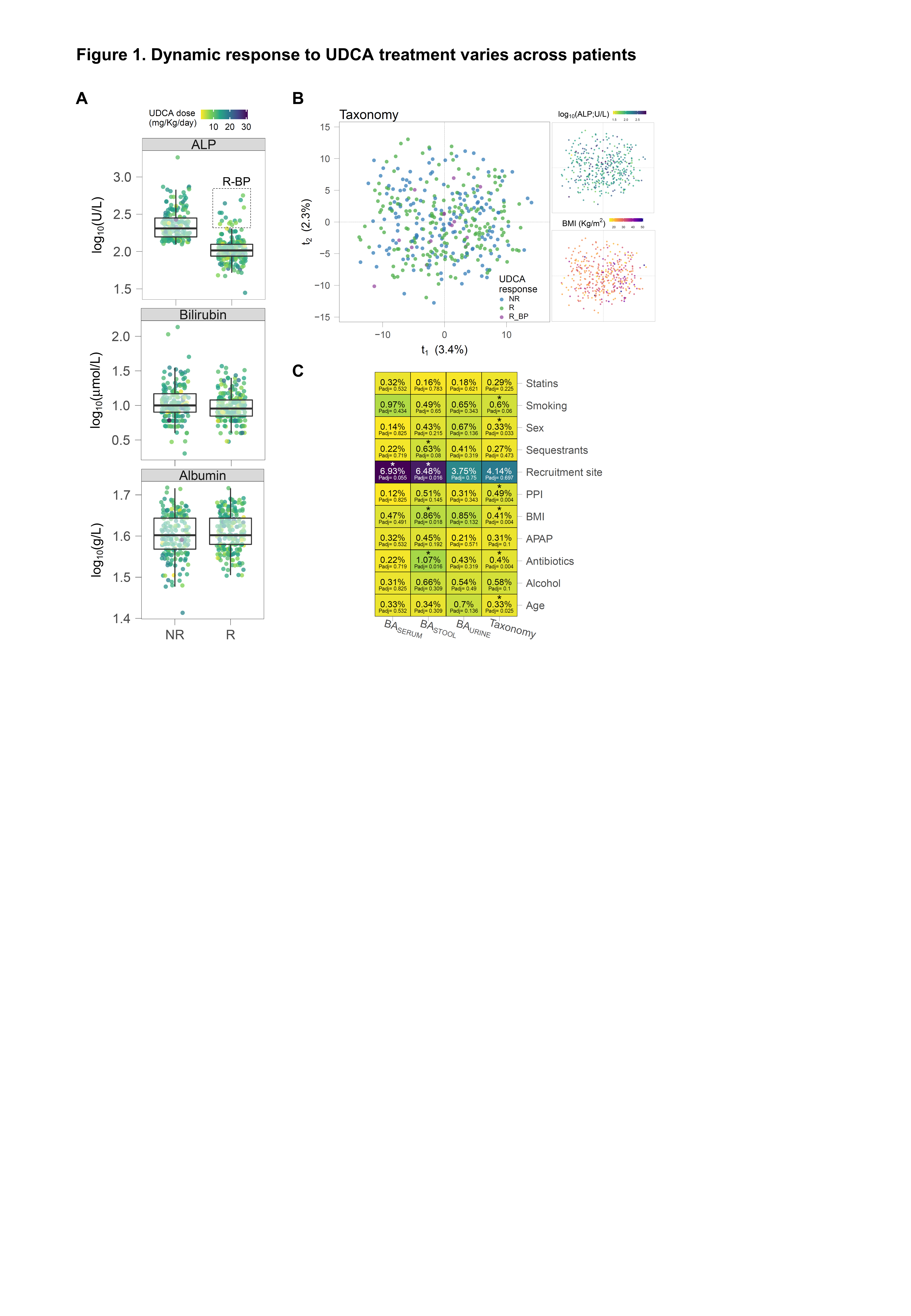

Supplement: Supplemental Material [file KGMI_A_2208501_SM5085.zip › KGMI_2208501_Supplemental/Supplementary_Figure1.PNG]

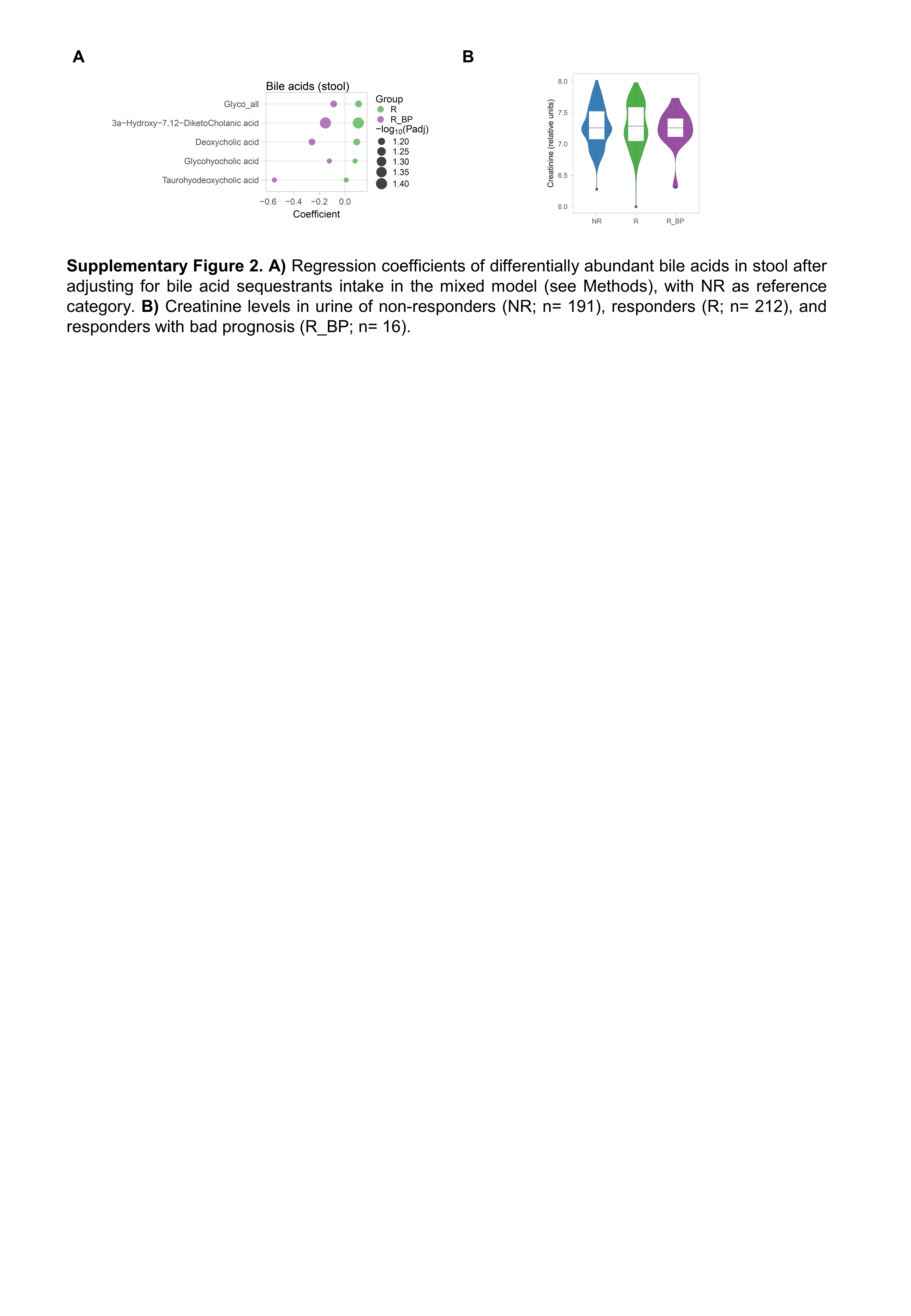

Supplement: Supplemental Material [file KGMI_A_2208501_SM5085.zip › KGMI_2208501_Supplemental/Supplementary_Figure2.PNG]

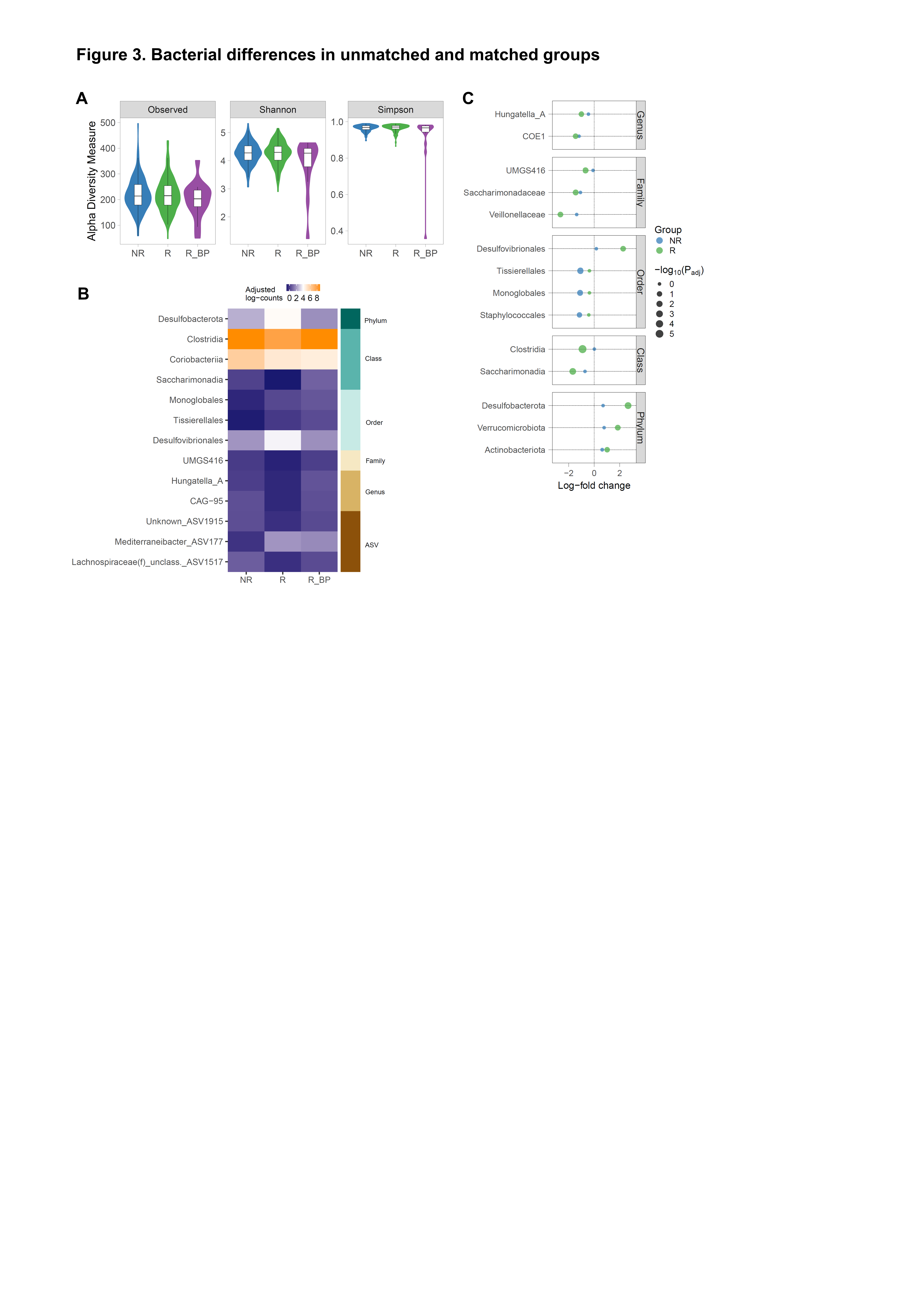

Supplement: Supplemental Material [file KGMI_A_2208501_SM5085.zip › KGMI_2208501_Supplemental/Supplementary_Figure3.PNG]

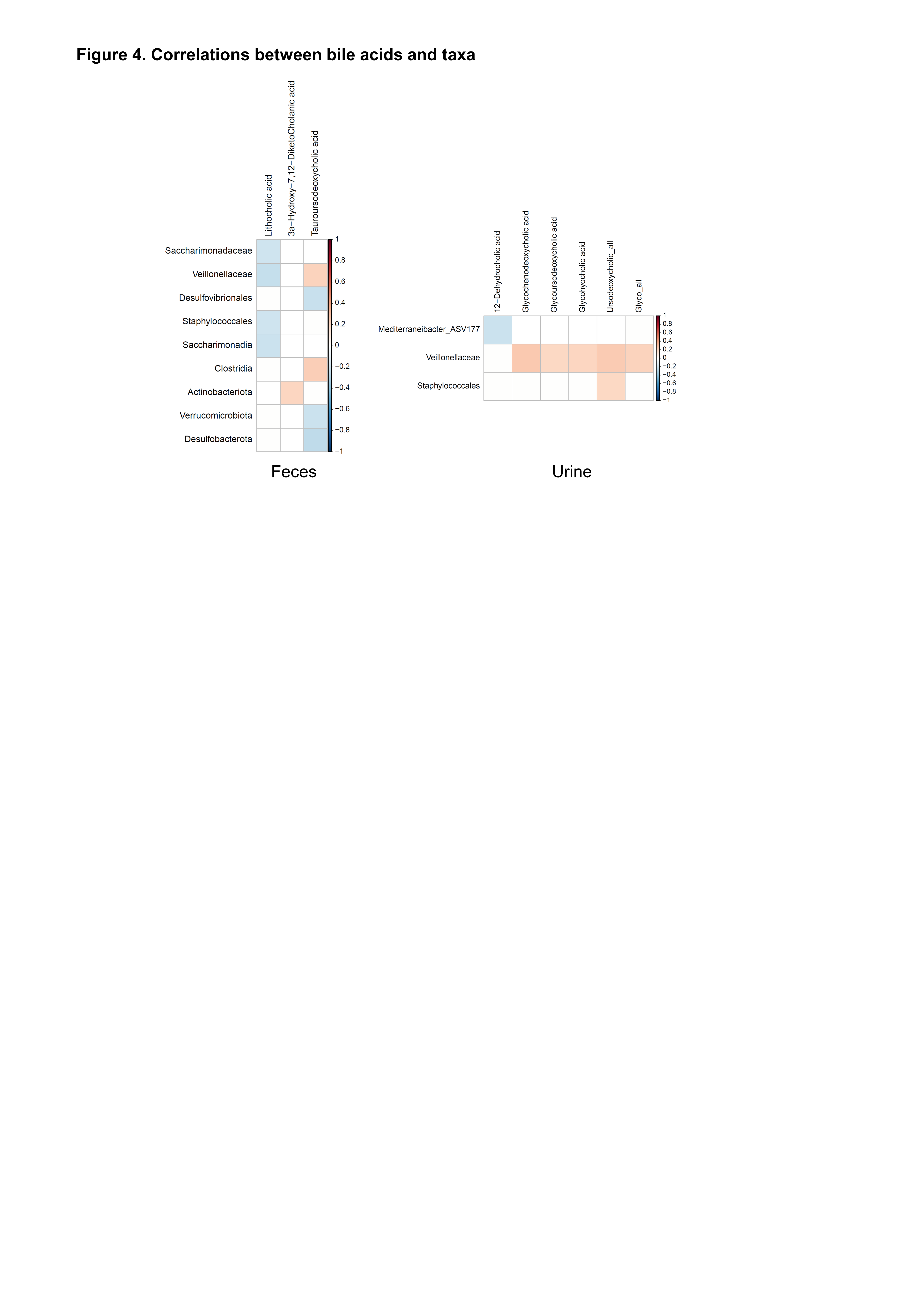

Supplement: Supplemental Material [file KGMI_A_2208501_SM5085.zip › KGMI_2208501_Supplemental/Supplementary_Figure4.PNG]

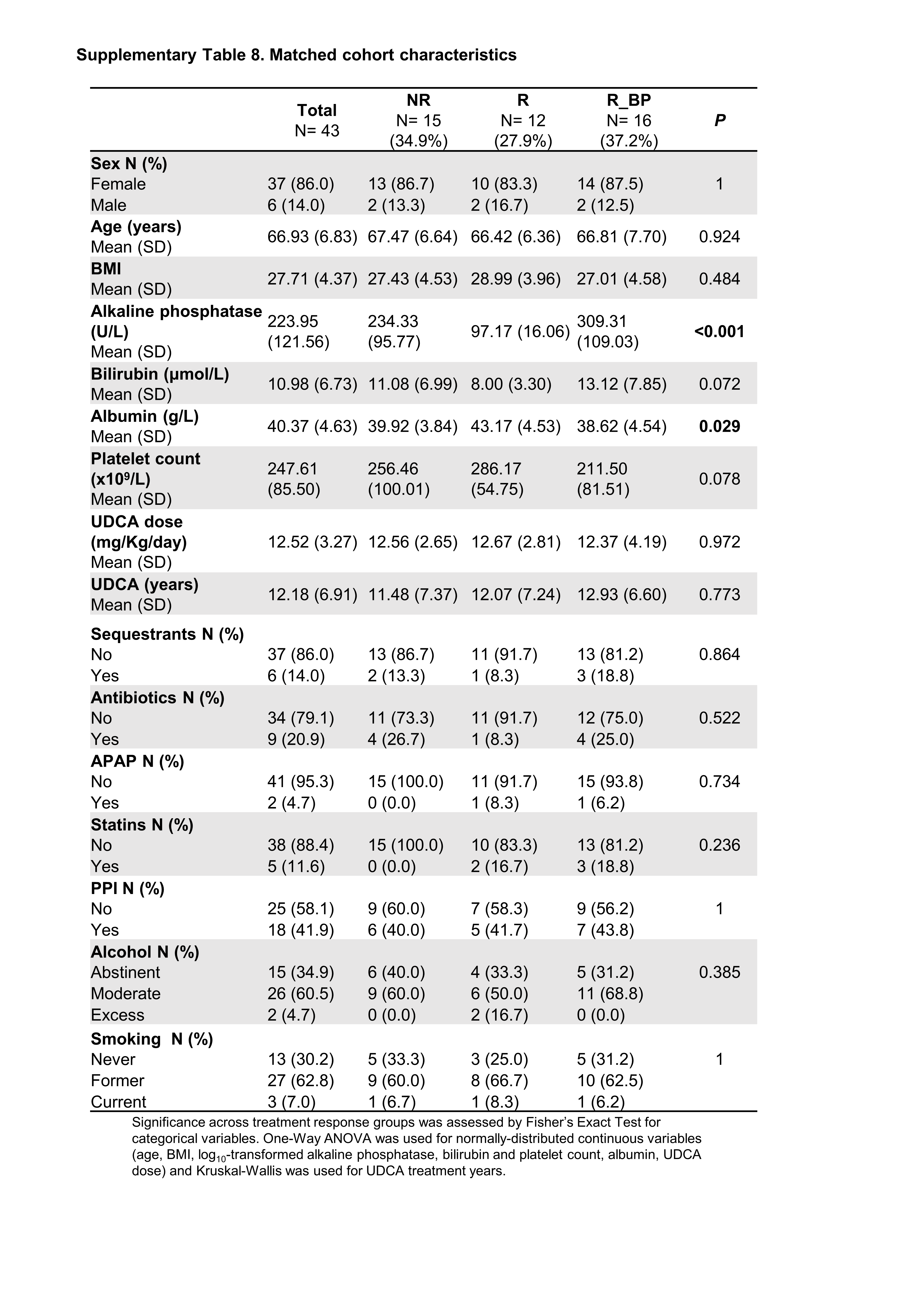

Supplement: Supplemental Material [file KGMI_A_2208501_SM5085.zip › KGMI_2208501_Supplemental/Supplementary_Table8.TIF]
